# Tomato *HAIRY MERISTEM* genes are involved in meristem maintenance and compound leaf morphogenesis

**DOI:** 10.1093/jxb/erw388

**Published:** 2016-11-03

**Authors:** Anat Hendelman, Michael Kravchik, Ran Stav, Wolfgang Frank, Tzahi Arazi

**Affiliations:** ^1^Institute of Plant Sciences, Agricultural Research Organization, The Volcani Center, 68 HaMaccabim Road, PO Box 15159 Rishon LeZion 7505101, Israel; ^2^Ludwig-Maximilians-Universität München, Department Biology I, Plant Molecular Cell Biology, LMU Biocenter, Grosshadernerstr. 2–4, D-82152 Planegg-Martinsried, Germany

**Keywords:** Cytokinin, HAM, leaf, meristem, miR171, tomato, WUSCHEL.

## Abstract

The *HAIRY MERISTEM* (*HAM*) genes function in meristem maintenance but play minor roles in the morphogenesis of a simple leaf that is determinate. Here, we functionally analyzed *HAM* genes in tomato and uncovered their involvement in compound leaf morphogenesis. Tomato encodes three *HAM* homologs, of which *SlHAM* and *SlHAM2* (*SlHAM*s) are guided for cleavage by microRNA171 and are abundant in the shoot and floral meristems as well as in the compound leaf primordia. We found that *SlHAM*s silencing led to overproliferation of cells in the periphery of the meristems where *SlHAM* is localized. As in meristems, leaf-specific silencing of *SlHAM*s provoked overproliferation of meristematic cells in the organogenic compound leaf rachis. We further demonstrate that the meristematic cell overproliferation in both meristems and leaves was in part due to the misexpression of the stem cell regulator *WUSCHEL*, previously shown to be induced by cytokinin. Strikingly, reduction of cytokinin levels in *SlHAM*s-silenced leaves completely suppressed the overproliferation phenotype, suggesting a regulatory link between *SlHAM*s and cytokinin, a key hormone found to promote indeterminacy in meristems and leaves. Taken together, our data provide evidence that in addition to their conserved function in meristem maintenance, *SlHAM*s are also required for the proper morphogenesis of the compound leaf.

## Highlight

Functional analysis of the tomato *HAM* genes suggest that they are involved in critical meristem functions and play homologous roles in compound leaf development.

## Introduction

The shoot apical meristem (SAM) produces all of a plant’s aboveground structures throughout its lifespan and it is also the site for the formation of new leaf and flower primordia. The SAM can be subdivided into a central zone (CZ) which consists of the stem cell niche at the SAM summit and the underlying organizing center (OC), a surrounding peripheral zone (PZ) of rapidly dividing cells, and an underlying rib zone (RZ). During organ formation, PZ and RZ cells are recruited into differentiating lateral organ primordia and the elongating stem, respectively (Steeves and Sussex, 1989). The ability of the SAM to maintain its size and continuously produce new organs depends on a small population of slowly dividing pluripotent stem cells in the CZ that constantly renews itself while providing daughter cells to the surrounding PZ and RZ (Tucker and Laux, 2007). *WUSCHEL* (*WUS*) is a central regulator of stem cell homeostasis and is specifically expressed in the meristem OC (Laux *et al.*, 1996; Mayer *et al.*, 1998). The floral meristem (FM) is a modified SAM, but, unlike the SAM, it is not indeterminate as its stem cells give rise to a precise number of floral organs and are abolished once the carpel primordia, the final organs to be made from the FM, form (Sablowski, 2007). Thus, a delicate balance between the two opposing activities of maintaining the undifferentiated stem cell population and promoting differentiation is precisely and carefully regulated in primary meristems.

The petunia *hairy meristem* mutant prematurely terminates both lateral organ and stem production due to precocious SAM termination. Terminating SAMs had a histological structure reminiscent of the radial pattern of the stem tissue subtending the wild-type SAM. This included the development of a differentiated epidermis with trichomes, hence the gene associated with the mutation designated *HAIRY MERISTEM* (*HAM*). Nevertheless, at termination, *hairy meristem* SAMs displayed normal *PhWUS* expression, suggesting that *PhHAM* is required for *PhWUS* responsiveness. *PhHAM* codes for a member of the GAI, RGA, and SCR (GRAS) family of transcription regulators (Bolle, 2004). It was concluded that *PhHAM* is required for meristem maintenance by protecting meristematic cells that initiate differentiation from developing by default into stem (Stuurman *et al.*, 2002).

The *Arabidopsis thaliana* (Arabidopsis) genome encodes four HAM (AtHAM)/LOST MERISTEM (LOM) homologs, of which *AtHAM1–AtHAM3* are targeted by miR171 (Llave *et al.*, 2002; Wang *et al.*, 2010). *Atham1,2* and *Atham1,2,3* mutants showed abrupt shoot termination and disturbed axillary bud formation, similar to *AtMIR171c* overexpressors and the *Phham* mutant (Stuurman *et al.*, 2002; Engstrom *et al.*, 2010; Schulze *et al.*, 2010; Wang *et al.*, 2010). The abrupt shoot termination was associated with an abnormal broader and flatter SAM that contained differentiating cells, indicating a reduced capacity to maintain itself and a loss of its polar organization (Schulze *et al.*, 2010). Occasionally, *Atham1,2,3* and *Atham1,2* mutants developed bulges or symmetrical organs in their leaf axils that contained ectopic meristematic cell clusters (EMCCs). EMCCs were also evident in the differentiated young stem (Schulze *et al.*, 2010). The blocked organogenesis despite ongoing cell division suggested that *AtHAM1* and *AtHAM2* are involved in the promotion of differentiation at the periphery of the SAM.

Recently, it was found that AtHAM1–AtHAM4 interact with WUS. The functionality of this interaction is supported by their co-localization in the SAM and the observation that the *Atham1,2,3* loss-of-function mutant and the *Atham4*-*pWUS:MIR171* mutant display defects similar to those observed in *wus7* and *wus1* mutants, respectively. In addition, AtHAM2 has been found to bind genomic regions similar to those reported to associate with WUS and to enhance WUS transcriptional activities. These data suggest that HAM proteins also function as cofactors for WUS-mediated stem cell niche maintenance (Zhou *et al.*, 2015).

In contrast to the significant effect of *ham* mutations on meristems, in Petunia and pepper *ham* mutants, leaf morphology could not be distinguished from that of the wild type (Stuurman *et al.*, 2002; David-Schwartz *et al.*, 2013). In Arabidopsis, loss of HAM functions caused narrower and more serrated rosette leaves and epinastic cauline leaves (Engstrom *et al.*, 2010; Wang *et al.*, 2010). These subtle leaf phenotypes suggest that as opposed to their essential function in meristems, *HAM* genes may play a minor role in the development of a simple leaf, which is a determinate organ.

Although determinate, specific regions of the leaf maintain transient intermediate growth. These include regions at the margin of the leaf primordium that may be patterned into a blade or any other marginal structures such as leaflets. Accordingly, these regions are active for short periods in the simple leaf primordium and remain active for longer periods in the compound leaf primordia that are elaborated by leaflets (Hagemann and Gleissberg, 1996). The mechanism that regulates leaf margin maintenance is not completely understood. To investigate whether *HAM* genes are involved in compound leaf development, we functionally analyzed *SlHAM* and *SlHAM2* from the compound leafed *Solanum lycopersicum* (tomato) via reverse genetics. This analysis suggests that the function of *SlHAM*s in meristem maintenance was also conserved in tomato. However, in contrast to the minor role of *HAM* genes in simple leaves, our analysis uncovers an essential role for *SlHAM* genes in compound leaf morphogenesis.

## Materials and methods

### Plant material and growth conditions

The tomato cv. M82 driver lines *35S:LhG4*, *FIL:LhG4*, *AP1:LhG4*, and *OP:AtCKX3* are described elsewhere (Shani *et al.*, 2010; Hendelman *et al.*, 2012). Tomato plants were grown under greenhouse conditions with temperatures ranging between 15 °C and 30 °C in a turf–peat mix with nutrients, using 4 liter pots. Germination and seedling growth took place in a growth chamber under a 16 h light, 8 h dark photoperiod (photosynthetic photon flux density: 50–70 μmol m^−2^ s^−1^) at a constant temperature of 24 °C. For crosses, the closed flowers of the corresponding homozygous responder line were emasculated by removal of the petals and stamens, and hand-pollinated with the pollen of the respective homozygous driver line.

### Sly-miR171 cleavage site mapping

The sly-miR171 cleavage site was mapped in mixed mRNA extracted from leaves, flower buds, and flowers at anthesis using a modified procedure of RLM-RACE (RNA ligase-mediated rapid amplification of cDNA ends) as described before (Hendelman *et al.*, 2012) with corresponding RACE and RACE-nested primers (for primer sequences, see Supplementary Table S1 at *JXB* online).

### Plasmid construction

For sly-MIR171a and sly-MIR171b reporter constructs, the sequences flanking pre-miR171a (SL2.50ch02:46752720..46753003) and pre-miR71b (SL2.50ch02:44783423..44783704) were PCR-amplified from tomato M82 genomic DNA with the primer pairs SlMIR171a_*Sal*I_F or SlMIR171b_*Sal*I_F and SlMIR171a_ *BamH*I_R or SlMIR171b_*BamH*I_R which contained *Sal*I and *Bam*HI sites at their 5' ends. After sequence verification, the amplified fragments were cloned into the *Xho*I/*Bam*HI sites of the pOp-TATA-BJ36 shuttle vector between an *OP* array (Moore *et al.*, 1998) and *Agrobacterium tumefaciens* octopine synthase terminator (OCS) to generate OP:SlMIR171a and OP:SlMIR171b constructs. The *Not*I fragments of these constructs were then mobilized into the binary vector pART27 to generate pART27-OP:SlMIR171a and pART27-OP:SlMIR171b.

### Transformation of tomato plants

The binary vectors pART27-OP:SlMIR171a and pART27-OP:SlMIR171b were transformed into tomato cv. M82 by co-cultivation of cotyledons derived from 14-day-old seedlings with *A. tumefaciens* strain GV3101 as described previously (Hendelman *et al.*, 2013).

### Total RNA extraction and sly-miR171 gel-blot analysis

Total RNA was extracted from different tomato tissues with Bio-Tri RNA reagent (Bio-Lab, Jerusalem, Israel) according to the manufacturer’s protocol. Sly-miR171 gel-blot analysis of total RNA was performed as described previously (Hendelman *et al.*, 2013) using a complementary radiolabeled oligonucleotide as a probe.

### Quantitative RT-PCR assays

First-strand cDNA was synthesized from 2 µg of total RNA with a Maxima first strand cDNA synthesis kit (Thermo Scientific, Vilnius, Lithuania) following the manufacturer’s instructions. A negative control (–RT) was used to ensure the absence of genomic DNA template in the samples. Three independent biological replicates were used for each sample, and quantification was performed in triplicate. PCR was performed in StepOnePlus (Thermo Scientific) following the manufacturer’s instructions. PCR products were analyzed using StepOne software version 2.2.2 (Thermo Scientific). Primers were designed around the corresponding sly-miR171 complementary site. The relative expression levels were calculated using the two-standard-curve method normalized to *SlTIP41* as the reference gene. Unless otherwise mentioned, statistically significant differences between samples were determined by Tukey–Kramer multiple-comparison test.

### SEM

For scanning electron microscopy (SEM) analysis, different plant tissues were collected and placed in FAA (3.7% formaldehyde, 5% acetic acid, 50% EtOH, v/v/v) solution until use. Then the FAA was removed and the tissues were washed in an increasing gradient of ethanol (up to 100%). Fixed samples were critical point dried, mounted on a copper plate, and gold coated. Samples were viewed in a Jeol 5410 LV microscope (Jeol, Tokyo, Japan).

### Histological analyses

For apices and leaves, tissues were collected and fixed with PFA [4% paraformaldehyde; 1× phosphate-buffered saline (PBS); 0.2% Tween-20 (v/v)] for 16 h. Then the samples were washed twice with 1× PBS and dehydrated in increasing concentrations of ethanol (10, 30, 50, 70, 80, 90, 95, and 100%). The samples were embedded with JB-4 (Electron Microscopy Sciences, Hatfield, PA, USA) according to the manufacturer’s protocol with slight modifications: after dehydration, the samples were incubated in the infiltration solution and placed at 4 °C in the dark under vacuum for 6 h (the infiltration solution was replaced twice). Polymerization was performed under anaerobic conditions. For flower analyses, tissues were collected and fixed in FAA until use, then dehydrated in increasing concentrations of ethanol, cleared with K-clear (Kaltek, Padova, Italy), and embedded in paraffin. Microtome-cut sections (10 µm and 2.5 µm thick for plastic and wax, respectively) were spread on microscope slides and stained with 0.1% (w/v) Safranin followed by 0.2% (w/v) Fast green (flowers) or 0.1% (w/v) Toluidine blue O (apices and leaves). Slides were examined under bright-field using an Olympus DP73 microscope equipped with a digital camera.

### In situ *hybridization*

Tissue fixation and *in situ* hybridization were performed as described previously (Hendelman *et al.*, 2013). Antisense probes were prepared as follows: *SlHAM* (*Solyc08g078800*), *H4* (*Solyc04g011390*), and *SlCLV3* (*Solyc11g071380*) cDNA sequences were amplified from M82 cDNA using corresponding primers and cloned into either pGEM-T easy (Promega, Madison, WI, USA) or pJet1.2 (Thermo Scientific). The *SlWUS* (*Solyc02g083950*) clone was kindly provided by Yuval Eshed (Weizmann Institute of Science). Following sequence and orientation verification, the probes were transcribed *in vitro* with MEGAscript T7 (Thermo Scientific) incorporating digoxigenin-11-UTP (Roch, Mannheim, Germany).

## Results

### The tomato miR171 (sly-miR171) guides the cleavage of three GRAS-like genes

Four members of the tomato miR171 family have been cloned to date, of which sly-miR171a and sly-miR171b are offset by three nucleotides relative to each other (Moxon *et al.*, 2008). Using the psRNATarget web server (Dai and Zhao, 2011) and applying a mismatch score of ≤2.0, we predicted four putative sly-miR171 targets from the available tomato gene models (ITAG release 2.30) (Supplementary Fig. S1A). Analysis of RNA from leaves, flower buds, and flowers by RLM-RACE confirmed sly-miR171-directed endonucleolytic cleavage of *Solyc08g078800* (*SlHAM*), *Solyc01g090950*, and *Solyc11g013150*. Sequencing of the amplified products indicated that *SlHAM* and *Solyc01g090950* are cleaved at positions that derived from targeting by sly-miR171a and sly-miR171b, whereas *Solyc11g013150* was cleaved at positions that indicated targeting by sly-miR171b only (Supplementary Fig. S1A). In contrast, the *Solyc02g085600* cleavage product was not recovered by us, consistent with its absence in published tomato degradome data (Karlova *et al.*, 2013), suggesting that it is not subject to significant sly-miR171-mediated cleavage.

Analysis of the predicted protein sequences of *SlHAM*, *Solyc01g090950*, *Solyc11g013150*, and *Solyc02g085600* revealed a similar overall architecture, including a variable N-terminus, which was much longer in *SlHAM* and *Solyc01g090950*, followed by a conserved GRAS domain and its characteristic VHIID, PFYRE, and SAW motifs (Pysh *et al.*, 1999) (Supplementary Fig. S1B). Phylogenetic reconstruction of their GRAS domains (Supplementary Fig. S1C) revealed that SlHAM, Solyc01g090950, and Solyc02g085600 belong to the HAM branch of GRAS (Bolle, 2004). The Solyc01g090950 protein was found to belong to a subclade that includes Arabidopsis HAM1, 2, 3, Petunia HAM, and SlHAM proteins (Stuurman *et al.*, 2002; Engstrom *et al.*, 2010) and, accordingly, was named SlHAM2. The Solyc02g085600 protein was found in the HAM subclade but shares the highest similarity with AtHAM4, which also does not undergo miR171-guided cleavage (Engstrom *et al.*, 2010), and accordingly was named SlHAM4. The Solyc11g013150 protein was not found in the HAM subclade but in a subclade of GRAS that contains the *Medicago truncatula* GRAS-like transcription factor NODULATION SIGNALING PATHWAY 2 (MtNSP2), which is targeted by mt-miR171h (Lauressergues *et al.*, 2012). In line with this, amino acid sequence alignment between Solyc11g013150 and MtNSP2 showed significant overall homology (62%/77% identity/similarity) and therefore it was named SlNSP2L (Supplementary Fig. S1C).

### *Silencing of* SlHAM*s in meristems is associated with the formation of EMCCs*

**Fig. 1.**
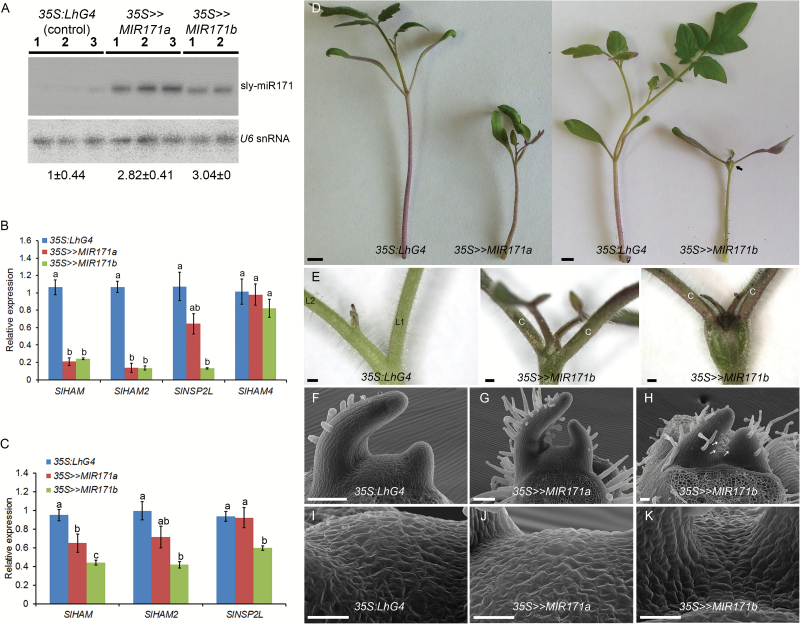
Phenotypic and molecular analyses of sly-miR171-overexpressing seedlings. (A) Northern blot analysis of sly-miR171 in 3-week-old control (*35S:LhG4*) and sly-miR171-overexpressing (*35S>>MIR171a*, *35S>>MIR171b*) seedlings. Total RNA was extracted from pooled independent seedlings (*n*=6 per biological replicate). Sly-miR171 expression levels were determined relative to the control after normalization to the *U6* snRNA and are indicated below each panel. (B, C) Quantification of sly-miR171 target mRNA levels in the RNA samples analyzed in A (B) and in apices of seedlings at 1.5 days after germination (DAG) (C) by qRT-PCR. Error bars indicate the SD over three biological replicates. The levels are expressed relative to the control, which was set to 1 ±SD. Different letters indicate statistically significant differences at *P*<0.01. (D) Seedlings from the indicated genotypes at 26 DAG. An arrow marks the bulged tissue. Scale bar=1 cm. (E) A close-up view of vegetative apices of 26 DAG seedlings from indicated genotypes. Two representative *35S>>MIR171b* apices with different bulges are shown. C, cotyledon; L1, first leaf; L2, second leaf. Scale bar=1 cm. (F–K) Scanning electron micrographs of shoot apices of 11 DAG seedlings from the indicated genotypes. The first leaf pair (F, G) and advanced leaf primordia (F–H) were removed. Arrows indicate representative trichomes on the adaxial side of the leaf primordium. (I–K) Magnified view of the meristem epidermis. Scale bars (F–H)=100 µm; (I–K)=25 µm.

**Fig. 2.**
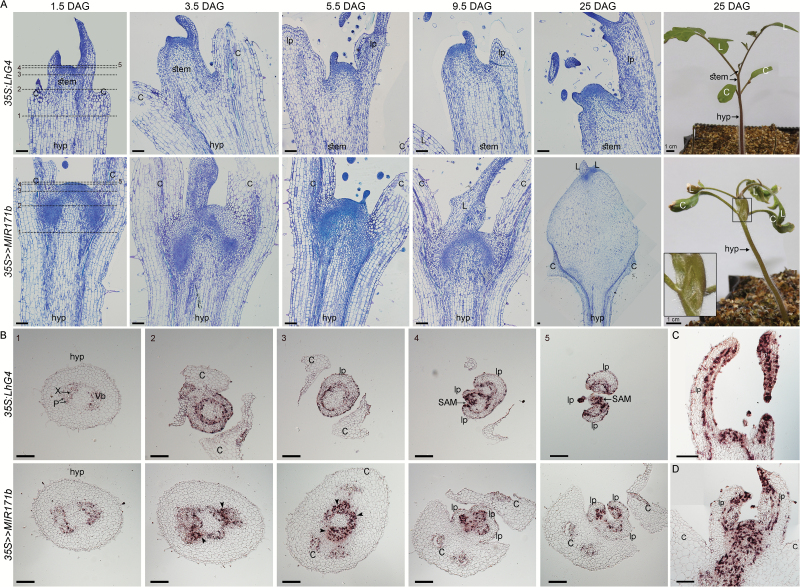
Histological analysis of *35S>>MIR171b* shoot apices. (A) A series of longitudinal sections of vegetative apices of seedlings at the indicated days after germination (DAG) stained with Toluidine blue. The lines mark the positions of the cross-sections shown in (B). The inset shows a magnified view of the bulging area. Note the fissures caused by the excess growth. (B–D) *In situ* localization of *histone H4* mRNA transcript in apices of seedlings at 1.5 DAG. (B) Successive transverse sections from the hypocotyl upward according to the indicated positions in (A). Arrowheads indicate clusters of proliferating cells. (C, D) Longitudinal sections. hyp, hypocotyl; C, cotyledon; X, xylem; P, phloem; vb, vascular bundle; L, leaf; lp, leaf primordium; SAM, shoot apical meristem. All scale bars=100 µm unless otherwise marked.

It was shown that HAMs from petunia, Arabidopsis, and pepper play a role in meristem maintenance (Stuurman *et al.*, 2002; Schulze *et al.*, 2010; David-Schwartz *et al.*, 2013; Zhou *et al.*, 2015). Consistent with this, published tomato RNA-sequencing data (Park *et al.*, 2012) have indicated that *SlHAM* and *SlHAM2* are relatively abundant in the SAM and FM. In contrast, in both meristems, *SlNSP2L* was expressed at ~11- to 15-fold lower levels and *SlHAM4* expression was almost negligible (~50- to 150-fold lower), suggesting that they are less important for meristem function (Supplementary Fig. S1D). To study the role of miR171-targeted *SlHAM* and *SlHAM2* (collectively referred to as *SlHAM*s) in tomato meristems, we utilized the OP/LhG4 transactivation system to silence them by overexpressing sly-miR171a, which directs the cleavage of both genes, or sly-miR171b that additionally cleaves *SlNSP2L* (Supplementary Fig. S2A). Following transformation into M82 tomato, 12 *OP:MIR171a* and five *OP:MIR171b* responder plants were obtained and their F_1_ progeny characterized for corresponding mature miR171 overexpression following a cross with the *35S:LhG4* driver line. This analysis identified several responder lines that strongly expressed the respective sly-miR171 upon transactivation (Supplementary Fig. S2B) from which we selected *OP:MIR171a-4* (hereafter *OP:MIR171a*) and *OP:MIR171b-20* (hereafter *OP:MIR171b*) for further analysis (Supplementary Fig. S2B). Constitutively activated *35S>>MIR171a* and *35S>>MIR171b* F_1_ progeny seedlings accumulated ~3-fold higher levels of corresponding sly-miR171 than the control (Fig. 1A) and that was accompanied by significantly lower levels of *SlHAM*s transcripts (Fig. 1B). A similar reduction in the levels of *SlNSP2L* was also observed, but only in *35S>>MIR171b* seedlings (Fig. 1B), in accordance with its specific cleavage by sly-miR171b (Supplementary Fig. S1A). Conversely, *SlHAM4* showed an insignificant reduction in its transcript levels, thus confirming its resistance to sly-miR171a- and sly-miR171b-guided cleavage in vivo (Fig. 1B). In contrast to whole seedlings, examination of *SlHAM*s levels in shoot apices of seedlings showed more efficient silencing in *35S>>MIR171b* than in *35S>>MIR171a*. In addition, *SlNSP2L* silencing was weaker than that of *SlHAM*s in *35S>>MIR171b* shoot apices. Whereas *SlHAM*s levels were down-regulated by ~55%, *SlNSP2L* levels were down-regulated by only 36% (Fig. 1C). Compared with the control (*35S:LhG4*), the *35S>>MIR171a* seedlings displayed growth arrest after producing a few pairs of leaves, suggesting that *SlHAM*s are required for normal meristem function (Fig. 1D). Consistent with the stronger silencing of *SlHAM*s in *35S>>MIR171b* seedlings, they displayed a similar, albeit more severe phenotype, and usually arrested growth after producing a single pair of abnormal leaves (Fig. 1D). In addition, they developed trichomes on the adaxial side of their arrested P2 leaf primordia, indicating precocious differentiation (Fig. 1F–H). However, unlike the Petunia *ham* mutant (Stuurman *et al.*, 2002), their SAMs did not display trichomes (Fig. 1I–K). Moreover, in two-thirds of the *35S>>MIR171b* seedlings, the shoot apex already appeared slightly swollen at 12 days after germination (DAG) and the swollen area enlarged over time to form a bulge between the cotyledons (Fig. 1E), which grew until it tore the surrounding tissue (Fig. 2A, 25 DAG). A similar phenotype was occasionally observed in *35S>>MIR171a* apices (Supplementary Fig. S3). The etiology and ultrastructure of the bulge were investigated by histology of young *35S>>MIR171b* seedlings and *histone H4* distribution, as a marker for cell division (Brandstädter *et al.*, 1993). In control tomato apices, high meristematic activity, as indicated by small densely Toluidine blue-stained cytoplasmic cells (Fig. 2A, *35S:LhG4*) and positive *histone H4* signal (Fig. 2B sections 4–5, and C), was restricted to the SAM and the adaxial side of the leaf primordia. In contrast, in *35S>>MIR171b* apices, ectopic clusters of small densely staining (Fig. 2A) and *histone H4*-positive (Fig. 2B sections 2–3, and D) cells were apparent between the cotyledons, indicating abnormal cell proliferation. With time, this led to the build up of a cell mass in the form of a bulge between the hypocotyl and the SAM (Fig. 2A, *35S>>MIR171b*). The apical part of the bulge consisted of small meristematic cell clusters that produced an inner mass of large and vacuolated cells, suggesting that as cells were displaced to the periphery they lost their meristematic nature without adopting a specific fate (Fig. 2A, 25 DAG).

**Fig. 3.**
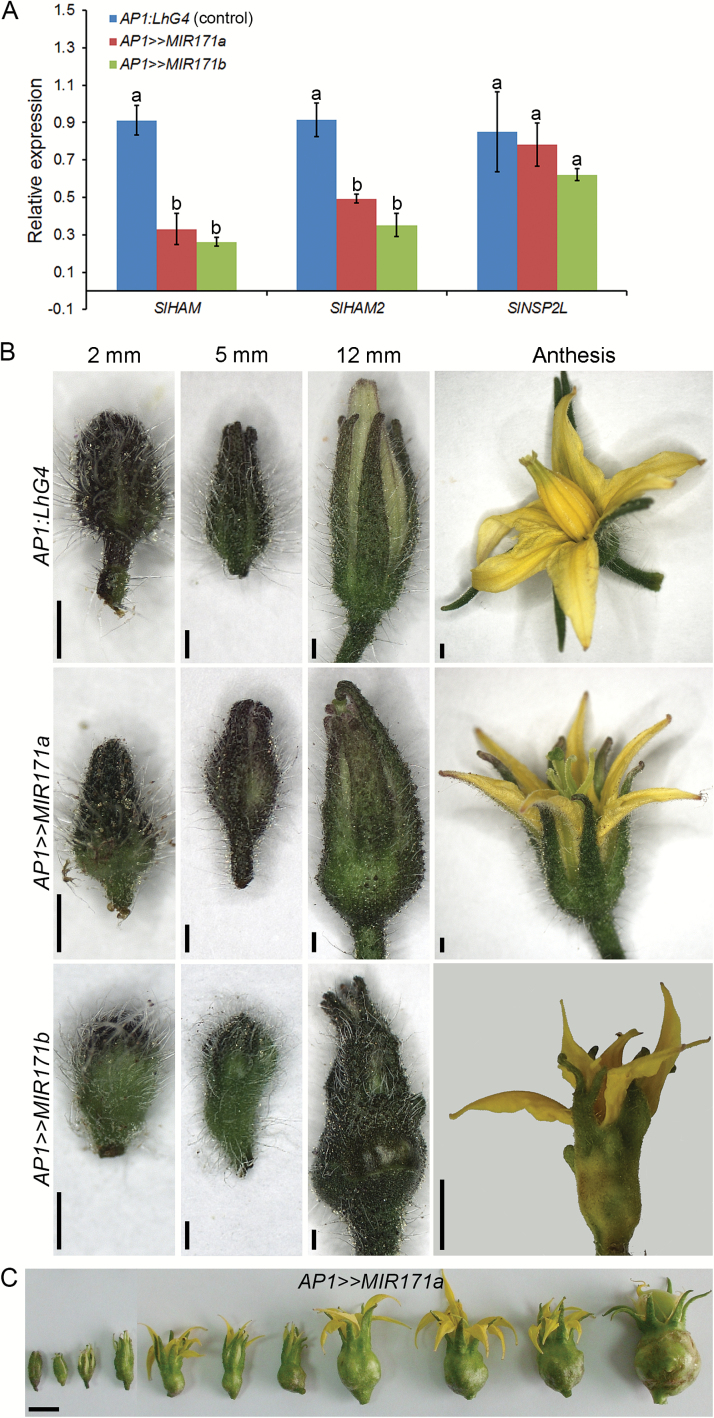
Silencing *SlHAM*s through the *AP1* promoter causes the formation of a bulge at the flower proximal end. (A) Quantification of *SlHAM*s and *SlNSP2L* mRNA levels in young inflorescences with buds <1 mm in size by qRT-PCR. Error bars indicate the SD over three biological replicates (*n*=25 per biological replicate). The levels are expressed relative to control, which was set to 1 ±SD. Different letters indicate statistically significant differences at *P*<0.01. (B) Photographs of floral buds at the indicated sizes. Scale bars=1 mm. (C) A development series of an *AP1>>MIR171a* flower. Note the gradual increase in the bulge size. Scale bar=1 cm.

**Fig. 4.**
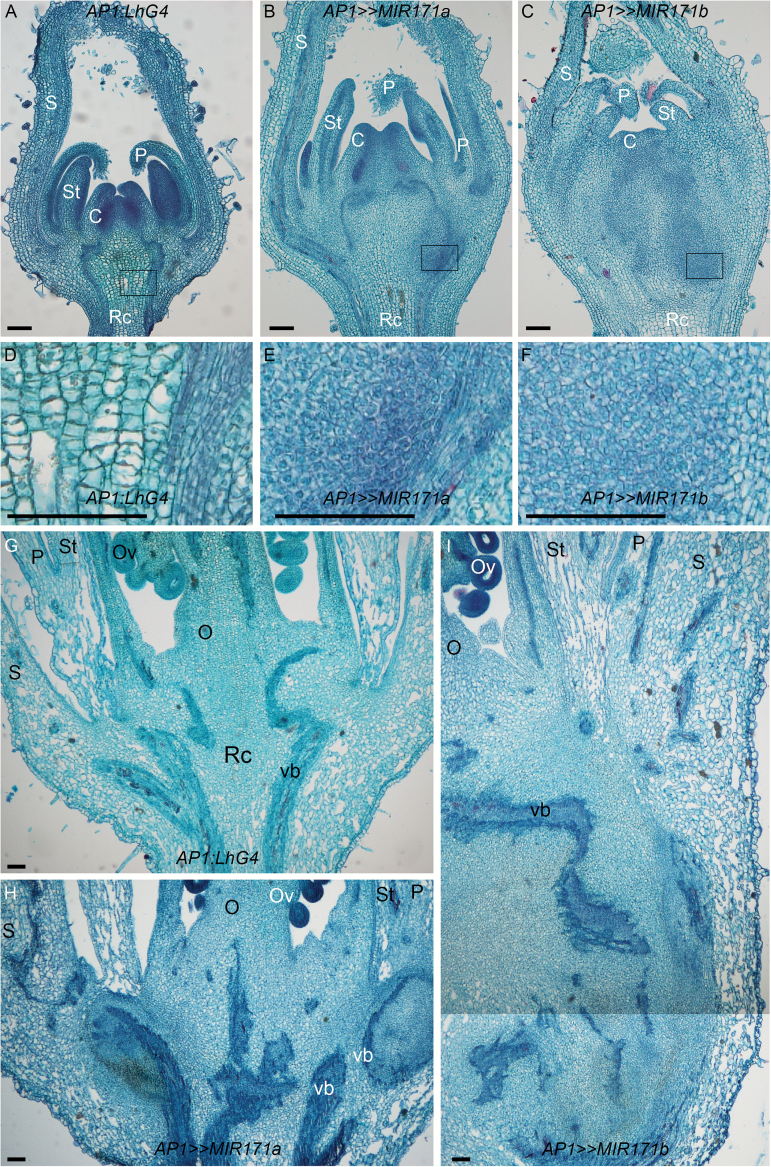
Histology of *AP1>>MIR171a* and *AP1>>MIR171b* flowers. (A–C) Longitudinal sections of 1.5 mm floral buds stained with Safranin and Fast-green. Representative densely stained areas are boxed. (D–F) Higher magnification of the respective boxed regions in (A–C). (G–I) Longitudinal sections of flowers at anthesis. Rc, receptacle; S, sepal; P, petal; St, stamen; O, ovary; Ov, ovule; C, carpel; vb, vascular bundle. All scale bars=100 µm.

Since *SlHAM* genes are also relatively abundant in the FM (Supplementary Fig. S1D), we examined their roles by reducing their expression levels specifically in the FM through crossing the *OP:MIR171a* and *OP:MIR171b* responder lines with the flower-specific *AP1:LhG4* driver line. *AP1:LhG4* drives expression throughout the FM and, when meristem activity is abolished, its expression is confined to sepals and petals (Hendelman *et al.*, 2012). Quantitation of sly-miR171-targeted genes in the young inflorescence with flower buds <1 mm in size revealed a significant ~65% reduction of *SlHAM* and *SlHAM2* in *AP1>>MIR171a* and *AP1>>MIR171b*, whereas no significant reduction in *SlNSP2L* levels was detected in these plants, which is consistent with its less efficient silencing in shoot apices (Fig. 3A). Phenotypic analysis of developing floral buds showed a similar abnormal morphology in *AP1>>MIR171a* and *AP1>>MIR171b*. The observed phenotype, which was more pronounced in *AP1>>MIR171b*, was already visible in 2 mm buds that displayed swollen proximal ends compared with *AP1:LhG4* control buds. Like the *35S>>MIR171b* seedling bulge (Fig. 2A), the swelling increased with time (Fig. 3B). In contrast to *AP1>>MIR171b* flowers, which senesce and could not bear fruit (Fig. 3B), the less severely affected *AP1>>MIR171a* buds reached anthesis and could set fruit. Moreover, the swollen area in the flower buds of *AP1>>MIR171a* plants continued to expand during fruit development, suggesting its indeterminate nature (Fig. 3C). Accordingly, histology of very young *AP1>>MIR171a* and *AP1>>MIR171b* flower buds revealed that bud swelling was caused by excess proliferation of cells above the receptacle, which was more dramatic in the *AP1>>MIR171b* buds (Fig. 4A–C). In contrast to the characteristic large vacuolated cells of the receptacle, the excess growth contained a mixed cell population that included numerous foci of relatively small densely stained cells (Fig. 4D–F), reminiscent of the actively dividing cell foci observed in the *35S>>MIR171b* apex (Fig. 2A, B) and the EMCCs observed in the *Atham1,2* mutant (Schulze *et al.*, 2010). Similar analysis of *AP1>>MIR171a* and *AP1>>MIR171b* flowers at anthesis revealed a larger tumor-like cell mass intertwined with numerous vascular bundles, demonstrating that as the flower matures, the respective cells continue to proliferate (Fig. 4G–I). Taken together, our data indicate that depletion of *SlHAM*s from the SAM and FM causes the formation of EMCCs in their periphery.

Since *SlHAM* genes are also relatively abundant in the FM (Supplementary Fig. S1D), we examined their roles by reducing their expression levels specifically in the FM through crossing the *OP:MIR171a* and *OP:MIR171b* responder lines with the flower-specific *AP1:LhG4* driver line. *AP1:LhG4* drives expression throughout the FM and, when meristem activity is abolished, its expression is confined to sepals and petals (Hendelman *et al.*, 2012). Quantitation of sly-miR171-targeted genes in the young inflorescence with flower buds <1 mm in size revealed a significant ~65% reduction of *SlHAM* and *SlHAM2* in *AP1>>MIR171a* and *AP1>>MIR171b*, whereas no significant reduction in *SlNSP2L* levels was detected in these plants, which is consistent with its less efficient silencing in shoot apices (Fig. 3A). Phenotypic analysis of developing floral buds showed a similar abnormal morphology in *AP1>>MIR171a* and *AP1>>MIR171b*. The observed phenotype, which was more pronounced in *AP1>>MIR171b*, was already visible in 2 mm buds that displayed swollen proximal ends compared with *AP1:LhG4* control buds. Like the *35S>>MIR171b* seedling bulge (Fig. 2A), the swelling increased with time (Fig. 3B). In contrast to *AP1>>MIR171b* flowers, which senesce and could not bear fruit (Fig. 3B), the less severely affected *AP1>>MIR171a* buds reached anthesis and could set fruit. Moreover, the swollen area in the flower buds of *AP1>>MIR171a* plants continued to expand during fruit development, suggesting its indeterminate nature (Fig. 3C). Accordingly, histology of very young *AP1>>MIR171a* and *AP1>>MIR171b* flower buds revealed that bud swelling was caused by excess proliferation of cells above the receptacle, which was more dramatic in the *AP1>>MIR171b* buds (Fig. 4A–C). In contrast to the characteristic large vacuolated cells of the receptacle, the excess growth contained a mixed cell population that included numerous foci of relatively small densely stained cells (Fig. 4D–F), reminiscent of the actively dividing cell foci observed in the *35S>>MIR171b* apex (Fig. 2A, B) and the EMCCs observed in the *Atham1,2* mutant (Schulze *et al.*, 2010). Similar analysis of *AP1>>MIR171a* and *AP1>>MIR171b* flowers at anthesis revealed a larger tumor-like cell mass intertwined with numerous vascular bundles, demonstrating that as the flower matures, the respective cells continue to proliferate (Fig. 4G–I). Taken together, our data indicate that depletion of *SlHAM*s from the SAM and FM causes the formation of EMCCs in their periphery.

### SlHAM*s are involved in compound leaf morphogenesis*

**Fig. 5.**
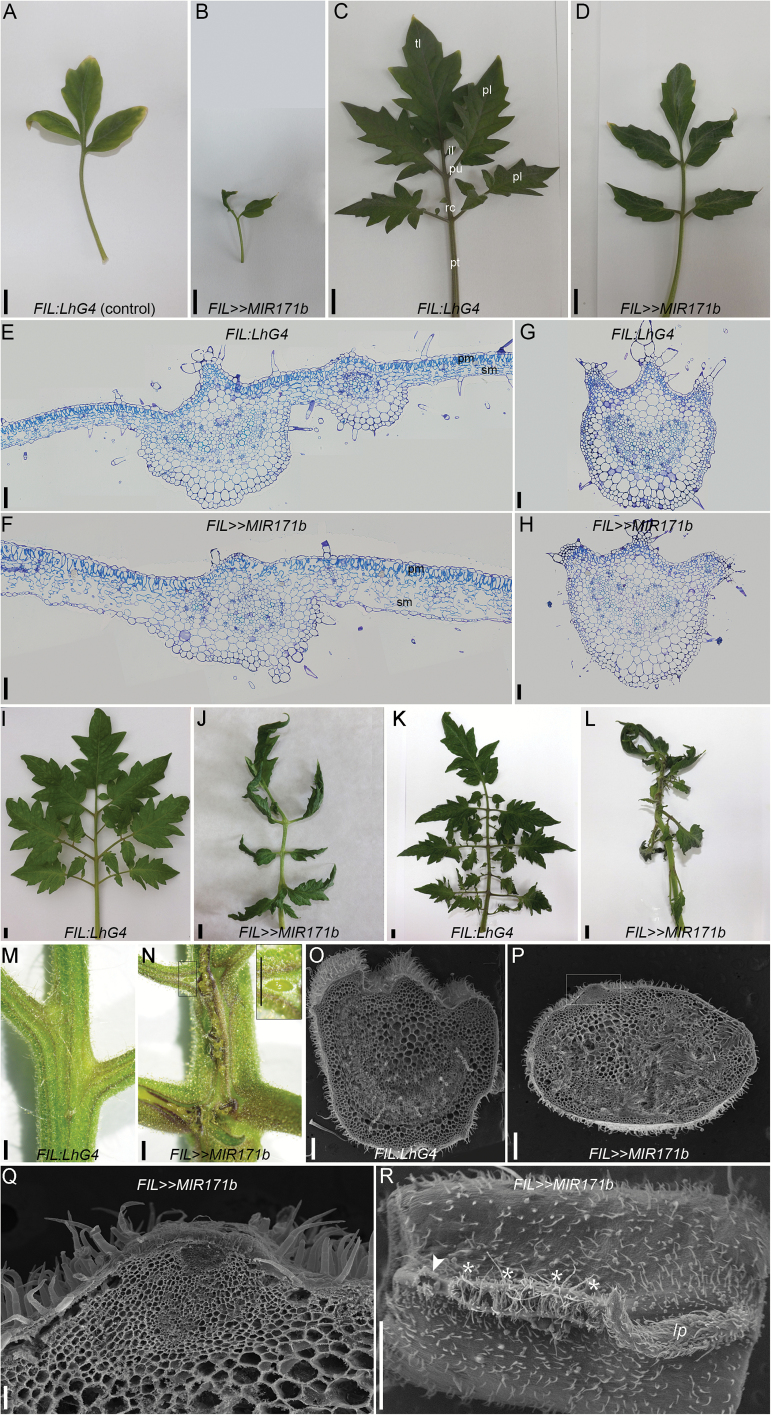
Characterization of *FIL>>MIR171b* leaves. (A–L) Representative leaves from the indicated genotypes. First leaf (A, B), fifth leaf (C, D), 10th leaf (I, J), >10th leaf (K, L). tl, terminal leaflet; pl, primary leaflet pair; il, intercalary leaflet; pu, petiolule; rc, rachis; pt, petiole. Scale bars=1 cm. (E–H) Cross-sections stained with Toluidine blue of the fifth leaf terminal leaflet (E, F) and rachis (G, H). pm, palisade mesophyll; sm, sponge mesophyll. Scale bars=100 µm. (M, N) Adaxial side of >10th leaf rachis. Note the ectopic growth protrusions in *FIL>>MIR171b*. Inset, close-up of the boxed region. Scale bars=1 mm. (O, P) SEM of a representative >10th leaf rachis cross-section. Scale bars=1 mm. (Q) Close-up of the inset boxed region in (P). Scale bar=100 µm. (R) SEM of the adaxial side of a >10th leaf rachis. Arrowhead and asterisks indicate bud and fasciated bud, respectively; lp, leaf primordium. Scale bars=1 mm.

We found that *SlHAM*s, which are relatively abundant in meristems, are also abundant in the compound leaf primordia, whereas *SlNSP2L* and *SlHAM4*, which were weakly expressed in the SAM and FM, maintained the same weak expression trend in the leaf primordia (Supplementary Fig. S1D). This led us to ask whether *SlHAM*s have a similar function in compound leaves. To investigate this, the *OP:MIR171b* responder line was crossed with the *FIL:LhG4* line driving transgene expression in lateral organ primordia but not in meristems (Shani *et al.*, 2009). Quantitation of sly-miR171-targeted genes in *FIL>>MIR171b* leaf primordia confirmed a significant reduction in the expression levels of *SlHAM*s compared with that of the control (Supplementary Fig. S4A). In the early developing leaves of *FIL>>MIR171b* plants, we observed relatively subtle phenotypes. Compared with the control *FIL:LhG4* line, these leaves were simpler and exhibited epinastic curling (Fig. 5A–D; Supplementary Fig. S4B). Histology of the fifth leaf indicated that the terminal leaflet blade is thicker than the control and contains more spongy mesophyll layers (Fig. 5E, F). In addition, both the leaflet and rachis displayed an atypical vasculature (Fig. 5E–H). Surprisingly, later developing leaves showed a dramatic increase in phenotype severity in *FIL>>MIR171b* plants. By leaf 10, the produced leaves were simpler, twisted, and their leaflet growth angle was distorted (Fig. 5I, J; Supplementary Fig. S4C). Later developing leaves were even more twisted and their leaflet growth angle was severely distorted. In addition, compared with the control, their rachis and attached petiolules were thicker and parts of their adaxial domain were abnormally brownish (Fig. 5K, L). As the leaves grew, a shiny light green tissue mass appeared on the adaxial side of the brownish rachis and petiolules (Fig. 5M, N). SEM of a cross-section of the *FIL>>MIR171b* brownish rachis revealed abnormal morphology and a bulge composed of relatively small cells at its adaxial side (Fig. 5O–Q). Moreover, in contrast to the hairy epidermis of control rachis, the epidermis above the bulge completely lacked trichomes (Fig. 5Q). The lack of trichomes and the presence of small cells instead of large cells suggest an undifferentiated meristematic nature of the ectopic bulging tissue. Microscopic examination of the adaxial side of the *FIL>>MIR171b* rachis confirmed its abnormal morphology and revealed a mixture of vegetative and reproductive lateral organ primordia that may have differentiated from the meristematic tissues (Fig. 5R). These observations suggest that in the organogenic compound leaf rachis, as in meristems, silencing of *SlHAM*s provokes overproliferation of meristematic cells.

### *The EMCCs in the* FIL>>MIR171b *leaves are suppressed by expression of a CK-degrading enzyme*

**Fig. 6.**
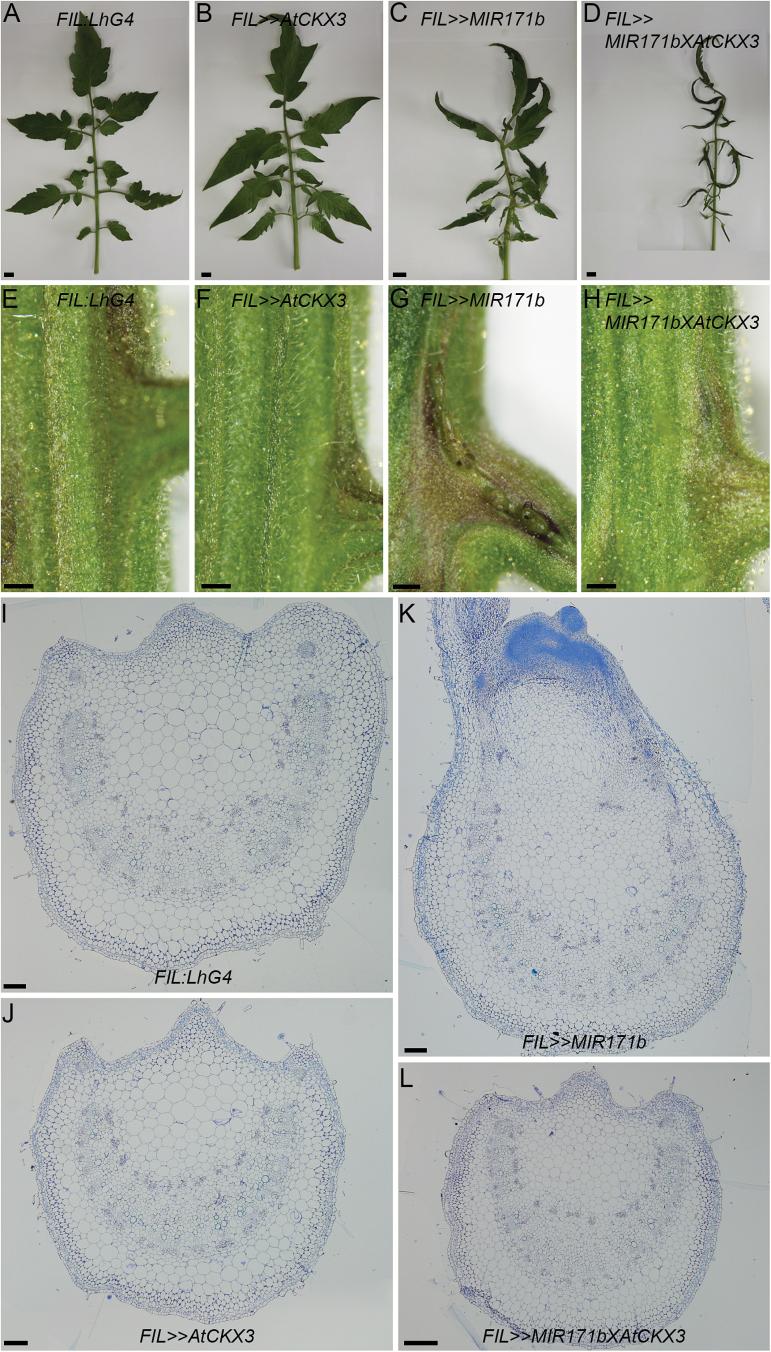
Restoration of the wild-type rachis phenotype upon expression of *AtCKX3* in *FIL>>MIR171b* leaves. (A–D) A representative 12th leaf from the indicated genotypes. Scale bars=1 cm. (E–H) Magnification of the adaxial side of the 12th leaf rachis. Note the ectopic growth protrusions in *FIL>>MIR171b* (G) and their absence in *FIL>>MIR171b>>AtCKX3* (H). Scale bars=500 µm. (K–M) Cross-sections stained with Toluidine blue of the 12th leaf rachis from the indicated genotypes. Scale bars=100 µm.

In tomato, elevation of cytokinin (CK) levels or response enhancement led to the proliferation of ectopic shoot and inflorescence meristems on the adaxial side of the compound leaf rachis which is highly reminiscent of the phenotype observed in *FIL>>MIR171b* later developed leaves (Janssen *et al.*, 1998; Kim *et al.*, 2003; Shani *et al.*, 2010; Steiner *et al.*, 2012). This suggested that an increased CK level or response may be the cause of the abnormal cell proliferation observed in later developed leaf rachises of *FIL>>MIR171b* plants. To test this idea, we reduced the CK levels in the *FIL>>MIR171b* leaves by expressing the Arabidopsis CK degradation gene *CYTOKININ OXIDASE3* (*AtCKX3*) (Werner *et al.*, 2003). To do this, *OP:MIR171b* was crossed to the previously characterized *OP:AtCKX3* responder line, which expresses *AtCKX3* upon transactivation (Shani *et al.*, 2010), and their double responder line progeny were crossed to the homozygous *FIL:LhG4* driver line. The leaf rachises of *FIL>>AtCKX3* tomato plants displayed a wild-type phenotype (Fig. 6B,F). In contrast to later developed leaves of *FIL>>MIR171b* which develop ectopic organ primordia on the adaxial side of their rachises due to abnormal proliferation of meristematic tissue (Fig. 6G, K), the leaves of *FIL>>MIR171b>>AtCKX3*-co-expressing *MIR171b* and *AtCKX3* under the *FIL* promoter displayed rachises that are morphologically identical to control and *FIL>>AtCKX3* leaves (Fig. 6D, H). In addition, histology of the *FIL>>MIR171b>>AtCKX3* leaf rachises confirmed that they possess wild-type morphology and are devoid of any abnormal clusters of densely stained meristematic cells, suggesting their suppression by a reduction of CK content (Fig. 6I–L). Still, similarly to *FIL>>MIR17b* leaves, *FIL>>MIR171b>>AtCKX3* leaflets exhibited epinastic curling (Fig. 6D), suggesting that this phenotype is not affected by CK content reduction.

### SlWUS *is misexpressed in the leaf rachis, SAM, and FM silenced for* SlHAM*s*

**Fig. 7.**
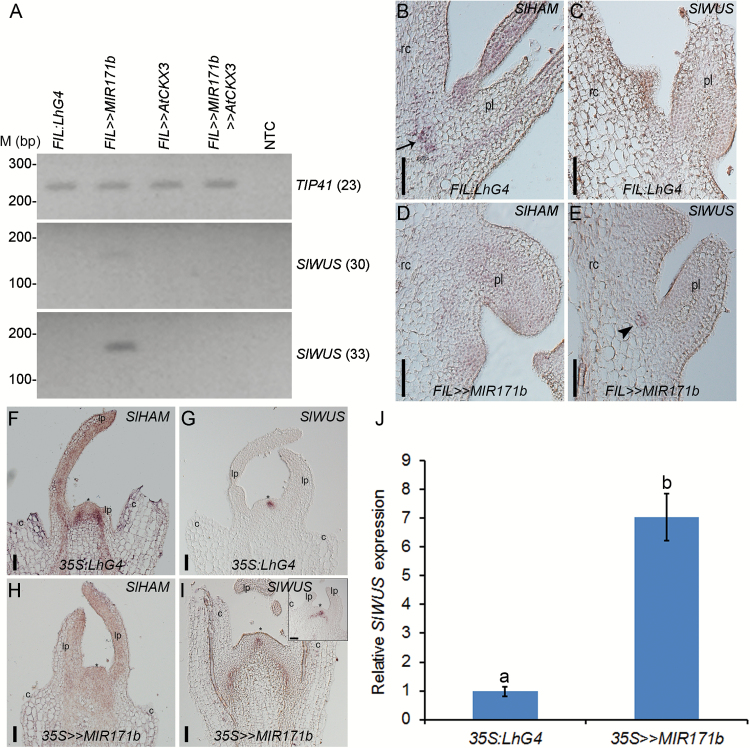
Misexpression of *SlWUS* upon *SlHAM*s silencing. (A) Semi-quantitative RT-PCR analysis of *SlWUS* in the rachis of the 10th leaf. The number of amplification cycles is indicated on the right. *SlTIP41* was used as a control. (B–E) Expression of *SlHAM* (B, D) and *SlWUS* (C, E) in leaf primordia at the P6 stage from 3-month-old *FIL:LhG4* (B, C) and *FIL>>MIR171b* (D, E) plants. *In situ* hybridization signals of *SlHAM* and *SlWUS* in the rachis are indicated by an arrow and an arrowhead, respectively. pl, primary leaflet; rc, rachis. Scale bars=50 µm. (F–I) *SlHAM* and *SlWUS* expression patterns were detected by *in situ* hybridization in apices of 1.5 DAG seedlings. The inset in (I) shows an additional apex with a milder phenotype. c, cotyledon; lp, leaf primordium; *, SAM. Scale bars=50 µm. (J) qRT-PCR analysis of *SlWUS* expression in 1.5 DAG apices of *35S:LhG4* and *35S>>MIR171b* plants. The same RNA was used as in Fig. 1C. Error bars indicate the SD over three biological replicates (*n*=20 per biological replicate). Different letters indicate statistically significant differences at *P*<0.01.

In Arabidopsis, CK is an important regulator of meristem establishment and maintenance by promoting *WUS* expression (Gordon *et al.*, 2009; Kuroha *et al.*, 2009; Bartrina *et al.*, 2011). Thus, to understand further how CK is involved in the overproliferation of meristematic cells in the *FIL>>MIR17b* rachis, we compared *SlWUS* expression in the 10th leaf rachis, which does not harbor visible ectopic meristematic tissue, between control, *FIL>>AtCKX3, FIL>>MIR17b*, and *FIL>>MIR171b>>AtCKX3*. In control and *FIL>>AtCKX3* rachises, we could not detect *SlWUS* by quantitative PCR (data not shown) or semi-quantitative RT-PCR, and similarly *SlWUS* was not detected by either in the rachises of *FIL>>MIR171b>>AtCKX3* leaves, corroborating their wild-type phenotype. Conversely, *SlWUS* was readily detected in *FIL>>MIR171b* rachises (Fig. 7A). Moreover, *in situ* hybridization detected *SlWUS* in the *FIL>>MIR171b* leaf rachis, but not in control leaf rachis that expressed *SlHAM* (Fig. 7B–E). This suggested that *SlHAM*s silencing in the leaf rachis caused *SlWUS* misexpression probably via CK. To test if *SlWUS* misexpression also occurs upon *SlHAM*s silencing in meristems, we compared the spatial distribution of *SlHAM* and *SlWUS* between control and *SlHAM*s-silenced SAM and FM. *In situ* hybridization on control vegetative apices showed that *SlHAM* transcripts accumulate in a pattern similar to that of *AtHAM1* (Schulze *et al.*, 2010). *SlHAM* accumulated mainly in the PZs from the L2 layer downward and in the rib meristem, but it was absent from the CZ that hosts the OC and stem cell niche (Fig. 7F). Instead of the characteristic OC-confined expression of *SlWUS* in the SAM of control plants (Fig. 7G), in *35S>>MIR171b* plants, the *SlWUS* expression domain expanded into the SAM periphery (Fig. 7I), probably causing the elevated *SlWUS* transcript levels detected by qRT-PCR (Fig. 7J). As in the SAM, non-overlapping expression domains of *SlHAM* and *SlWUS* were evident in the FM of control plants (Supplementary Fig. S5C, E) while *SlWUS* expression domains expanded in the FM of *FIL>>MIR171b* plants (Supplementary Fig. S5D, F). This suggests that similar to the leaf rachis, silencing of *SlHAM*s in the SAM and FM is correlated with *SlWUS* misexpression. In agreement with *WUS* being a positive regulator of stem cells and *CLV3* being a stem cell marker (Fletcher *et al.*, 1999), an ectopic *SlCLV3* expression was observed in the leaf rachis, SAM, and FM silenced for *SlHAM*s (Supplementary Fig. S6). Thus, in the absence of *SlHAM*s, misexpression of *SlWUS* led to the formation of extra stem cells that most probably caused the EMCCs observed in the SAM, FM, and the leaf rachis.

## Discussion

The *HAM* genes encode GRAS transcriptional regulators that have been implicated in meristem maintenance but were found to play only minor roles in simple leaf development (Stuurman *et al.*, 2002; Schulze *et al.*, 2010; Engstrom *et al.*, 2010; David-Schwartz *et al.*, 2013; Zhou *et al.*, 2015). In this study, we used global and tissue-specific trans-silencing to analyze *SlHAM*s functions in tomato that have compound leaves. A common phenotype of trans-silenced meristems and compound leaves was the formation of an ectopic cell mass as a result of overproliferation of meristematic cells.

Does *SlNSP2L* contribute to the observed overproliferation phenotypes? Despite *SlNSP2L* targeting by sly-miR171b, our analyses indicate that its silencing in vegetative and reproductive apices, which overexpress sly-miR171b, was weaker than that of *SlHAM*s (*35S>>MIR171b*; Fig. 1C) or not significant (*AP1>>MIR171b*; Fig. 3A), suggesting a minor or no contribution, respectively, to their abnormal phenotypes. The reduced silencing efficiency of *SlNSP2L* compared with *SlHAM*s may be caused due to the presence of an additional mismatch with in the seed sequence of sly-miR171b (Supplementary Fig. S1A). Moreover, cell proliferation phenotypes were also observed in *35S>>MIR171a* and in *AP1>>MIR171a* apices, which contained wild-type *SlNSP2L* levels (Figs 1C, 3A), indicating that its presence cannot rescue these phenotypes, probably because its function is not redundant with that of the silenced *SlHAM*s. This conclusion is also supported by the relatively weak expression of *SlNSP2L* in the SAM, FM, and leaf primordium (Supplementary Fig. S1D) and the absence of abnormal cell proliferation phenotypes in known *nsp2* mutants (Kaló *et al.*, 2005; Liu *et al.*, 2011). In contrast to *SlNSP2L*, *SlHAM*s were abundant in the SAM and FM, and *SlHAM* transcript accumulated mainly in the PZ and was highly reduced or excluded from the CZ. This pattern of transcript accumulation in meristems was very similar to that of *HAM* genes from Petunia, pepper, and Arabidopsis, suggesting a conserved function for *SlHAM*s in tomato meristems (Stuurman *et al.*, 2002; Schulze *et al.*, 2010; David-Schwartz *et al.*, 2013).

The ability of the SAM to produce new organs requires a delicate balance between maintenance of the indeterminacy potential and organogenesis. All known *ham* mutants ceased organ formation after producing a variable number of leaves as a result of precocious termination of the SAM (Stuurman *et al.*, 2002; Engstrom *et al.*, 2010; Schulze *et al.*, 2010; David-Schwartz *et al.*, 2013). This indicated that *HAM* genes are required to maintain SAM activity, and several roles have been proposed. In the Petunia *ham* mutant, the SAM differentiated into a stem two weeks after termination, suggesting that *PhHAM* is required for meristem maintenance by protecting meristematic cells that initiate differentiation from developing by default into stem (Stuurman *et al.*, 2002). In Arabidopsis, AtHAMs were recently demonstrated to be important for stem cell production by physically interacting with WUS and facilitating its related transcriptional activities. Accordingly, similar to *wus* mutants, *Atham* mutants show an arrested growth phenotype probably due to precocious termination of their stem cell-depleted SAMs. In tomato, physical interaction between *SlHAM* and *SlWUS* suggests a similar function in stem cell homeostasis for the former (Zhou *et al.*, 2015). This is supported by early vegetative growth arrest of *SlHAM*s-silenced seedlings and the direct correlation between the silencing efficiency and growth arrest severity. While the *35S>>MIR171b* apices arrested just after producing two leaves, the *35S>>MIR171a* apices, which contained higher *SlHAM*s levels, produced more leaves (Fig. 1C, D). However, since *SlHAM*s-silenced seedlings contained EMCCs already at 1.5 DAG, it is hard to determine the cause and effect of their growth arrest.

In shoot meristems, *AtHAM1* and *AtHAM2* have also been suggested to promote cellular differentiation of CZ descendants. This is supported by their preferred accumulation in the SAM PZ, the blocked organogenesis, and the development of EMCCs in the axils and young stems of corresponding mutants. Moreover, the EMCCs accumulated the transcripts of *WUS* and the stem cell marker *CLV3*, and occasionally they proliferated to produce an axillary bulge instead of an organ (Schulze *et al.*, 2010). In tomato, *SlHAM*s accumulate in the meristem PZ, and their silencing in the SAM and FM was also associated with EMCCs that produced bulges beneath the corresponding meristem. In addition, formation of EMCCs was associated with expansion of *SlWU*S and *SlCLV3* expression domains. Taken together, these observations support a similar role for *SlHAM*s as promoters of cellular differentiation in meristems.

Interestingly, we also observed the formation of EMCCs associated with *SlWUS* and *SlCLV3* misexpression in the adaxial side of the compound leaf rachis, the site from which leaflet primordia emerge, and meristematic potential persists due to the activity of a pool of undifferentiated cells at the leaf margin (Hagemann and Gleissberg, 1996; Kim *et al.*, 2003). It was suggested that once lobes and leaflets are initiated in the tomato compound leaf, this meristematic activity becomes restricted to those structures and down-regulated along the rachis (Kang and Sinha, 2010). This raises the possibility that as in meristems, *SlHAM*s may promote the differentiation of the rachis cells in order to prevent excess cell proliferation that could lead to the development of ectopic lateral organs. In tomato, leaf complexity increases acropetally, suggesting that the early developed leaf rachis maintains its meristematic potential for a shorter period than the later developed one (Busch *et al.*, 2011). This provides a possible explanation for why EMCCs were never observed in the rachises of early developing leaves. In these rachises, the meristematic activity probably arrested before their cells could proliferate to significant numbers to generate an EMCC. Accordingly, meristematic cell proliferation has never been reported in the previously characterized *ham* mutants (Stuurman *et al.*, 2002; Engstrom *et al.*, 2010; Wang *et al.*, 2010; David-Schwartz *et al.*, 2013), probably due to the relatively rapid differentiation of their simple leaves. Instead, their simple leaves showed no phenotype (Stuurman *et al.*, 2002; David-Schwartz *et al.*, 2013) or relatively subtle phenotypes, including an increase in cell number along the adaxial–abaxial axis of the leaf and epinastic curling (Engstrom *et al.*, 2010; Wang *et al.*, 2010). The latter are reminiscent of the phenotypes observed in *FIL>>MIR171b* leaflets, suggesting that *HAM* genes also play a role in blade development.

We have shown that leaf-specific expression of *AtCKX3*, a CK-degrading enzyme, did not rescue blade development but suppressed the formation of EMCCs in *FIL>>MIR171b* leaf rachises, suggesting that EMCCs are CK dependent. Consistent with that, in tomato, increased CK levels or signaling cause the formation of ectopic meristems on the adaxial side of the leaf rachis and leaflet petiolules (Shani *et al.*, 2010; Steiner *et al.*, 2012). Since CK was shown to promote *WUS* expression in meristems (Gordon *et al.*, 2009), the expansion of the *WUS* domain in meristems silenced for *SlHAM*s may imply that CK status increased in these meristems. This may hold for *SlHAM*-silenced leaf rachises since *SlWUS* was also misexpressed in them. Taken together, our results raise the possibility that by negative regulation of CK levels in the SAM, FM, and the compound leaf margin, either directly or indirectly, *SlHAM*s may promote cellular differentiation in them. Further research is required to confirm this hypothesis.

## Supplementary data

Supplementary data are available at *JXB* online.

Table S1. Primers and probes used in this study.

Figure S1. Characterization of sly-miR171 predicted target genes.

Figure S2. Generation and screening of transgenic tomato responder lines.

Figure S3. Phenotype of *35S>>MIR171a* seedlings 25 DAG.

Figure S4. Characterization of *FIL>>MIR171b* leaves.

Figure S5. *In situ* hybridization of *SlHAM* and *SlWUS* in *FIL>>MIR171b* floral buds.

Figure S6. *In situ* hybridization of *SlCLV3* in *SlHAM*-silenced plants.

Supplementary Data
