# Supplementary material for: Tomato HAIRY MERISTEM genes are involved in meristem maintenance and compound leaf morphogenesis
Source: J Exp Bot. 2016 Nov 3;67(21):6187–200. doi: 10.1093/jxb/erw388 (PMC5100029; doi:10.1093/jxb/erw388)
Supplement: Supplementary Data [file supp_67_21_6187__index.html]

Tomato HAIRY MERISTEM genes are involved in meristem maintenance and compound leaf morphogenesis — Supplementary Data 

# Tomato *HAIRY MERISTEM* genes are involved in meristem maintenance and compound leaf morphogenesis

## Supplementary Data

Data files

- supplementary\_table\_S1\_figures\_S6.pdf - Supplementary Data
