# Supplementary material for: Tomato HAIRY MERISTEM genes are involved in meristem maintenance and compound leaf morphogenesis
Source: J Exp Bot. 2016 Nov 3;67(21):6187–200. doi: 10.1093/jxb/erw388 (PMC5100029; doi:10.1093/jxb/erw388)

The tomato HAIRY MERISTEM genes are involved in meristem maintenance and compound leaf morphogenesis

Anat Hendelman, Michael Kravchik, Ran Stav, Wolfgang Frank and Tzahi Arazi

Supplementary data

Table S1. Primers and probes used in this study.

Supplementary Fig. S1. Characterization of sly-miR171 predicted target genes.

Supplementary Fig. S2. Generation and screening of transgenic tomato responder lines.

Supplementary Fig. S3. Phenotype of *35S>>MIR171a* seedlings 25 DAG.

Supplementary Fig. S4. Characterization of *FIL>>MIR171b* leaves.

Supplementary Fig. S5. In-situ hybridization of *SlHAM* and *SlWUS* in *FIL>>MIR171b* floral buds.

Supplementary Fig. S6. In-situ hybridization of *SlCLV3* in *SlHAM*s-silenced plants.

Table S1.

Supplementary Table 1. Primers and probes used in this study			
**Primer ID**	**Primer sequence (5' - 3')[a]**	**Gene ID**	**Remarks**
GeneRacer-5'	CGACTGGAGCACGAGGACACTGA		5'RACE
GeneRacer-5'_Nested	GGACACTGACATGGACTGAAGGAGTA		
5U600069_Nested	GCTGCAAGTACATCTCCCTGACGGTGTTC	*Solyc11g013150*	
5U600069_Race	TGCTGTGATCCGAAGATGCGGAGTTG		
5U569651_RACE	CGCTGCTGCCATGAGAAGGTGAACA	*Solyc01g090950*	
5U569651_Nested	ACCGGCGACCAGTCATTCCATGAAG		
5U592620_Race	CGTGAATGGCTCCAGCTCCGTCACTA	*Solyc08g078800*	
5U592620_Nested	GCACGACGAAGATGAAGCTGAAGGACTTG		
5U567228-Race	TCGAGCACTGACTTAGGTTCG	*Solyc02g085600*	
5U567228-Nested	GGGCAGGGATGGGAGGGTGCATTAT		
sly-miR171a_RC	GATATTGGCACGGCTCAATCA		Transgene identification and miR probes
sly-miR171b_RC	CGTGATATTGGCACGGCTCAA		
U6_RC	AGGGGCCATGCTAATCTTCTC		U6 snRNA probe
SlMIR171a_SalI_F	CGCGTCGACTCTCCCTCTTTTAG		Plasmid construction
SlMIR171a_BamHI_R	CGGGATCCACCCTAGATGGTAATGC		
SlMIR171b_SalI_F	CGCGTCGACATCCTCCCATCC		
SlMIR171b_BamHI_R	CGGGATCCAGGAAAGGGCGG		
OP1035SF	TCTTCGCAAGACCCTTCCTCTAT		Transgene identification
OCS_rev	GAAACCGGCGGTAAGGATCT		
U60069_KPNI_F	GGGGTACCATGGCTATGATGGTTGATGAAACC		
qRT-SGN-U600069_R	TGTAACGTACCGGAAGCCATCA		
qRT_SlWUS_F	GGTTATGGAACTTTGGCTATGGAG	*Solyc02g083950*	Real time primers
qRT_SlWUS_R	GAAAAGGGTAAGTTGCTGGAGAAG		
qRT-SGN-U567228_F	TTCGAAACAAACGCTTTCCAAC	*Solyc02g085600*	
qRT-SGN-U567228_R	GCAGCTCGTTGGAGAGGTTTTC		
qRT-SGN-U569651_F	TGTTGGTGTTGGCATGGAGTAGCA	*Solyc01g090950*	
qRT-SGN-U569651_R	GCAGAGCTGGTCCAGACAGGGA		
qRT-SGN-U592620_F	TTCAGGCCTCTGAACTATTGCT	*Solyc08g078800*	
qRT-SGN-U592620_R	CAACTGCAGAGCCTCCTTGATA		
qRT-SGN-U600069_F	GCGGAGGCATTAACCGGCGT	*Solyc11g013150*	
qRT-SGN-U600069_R	TGTAACGTACCGGAAGCCATCA		
qRT-TIP41_F	ATGGAGTTTTTGAGTCTTCTGC	*Solyc10g049850*	
qRT-TIP41_R	GCTGCGTTTCTGGCTTAGG		
PGEM-T7	TAATACGACTCACTATAGGG		In situ
WUS-InSt-F	AAGTAGTAGCCGTTGGACGC	*Solyc02g083950*	
SlHAM-FL-Cla-inst_F	AATATCGATATGATTGTAATACCTCAAAGTA	*Solyc08g078800*	
SlHAM-FL-Bgl-inst_R	ACCAGATCTGCACCTCCAAGTCACAGC		
H4-inst_F	GGGTATCACCAAGCCTGCTA	*Solyc04g011390*	
H4-inst_R	AACCACCGAACCCATAGAGAG		
clv3_NdeI_inst_F	ATTCCATATGATGTCTTTGATCAATGCTAAATA	*Solyc11g071380*	
clv3_NcoI_inst_R	AAACCATGGGGTTTCTTAGGACTAGCACCATTC		

[a]Sequences corresponding to restriction enzyme sites are underlined.

Fig. S1. Characterization of sly-miR171 predicted target genes. (A) Experimental validation of sly-miR171 cleavage site. Alignment between sly-miR171a (red) and sly-miR171b (blue) and their predicted target mRNAs. The arrows and numbers indicate the positions of the cleavage sites inferred from RLM-RACE and the fraction of sequenced clones, respectively. (B) Scheme of their predicted protein structure. In each protein, the relative locations of the GRAS domain and the conserved motifs are shown. The predicted protein length is indicated on the right. (C) An unrooted phylogenetic tree of the GRAS domains from Arabidopsis and selected tomato proteins, petunia HAM (PhHAM) and Medicago NSP2. The tree was produced by neighbor-joining with 100 bootstrap sampling (MEGA program, version 4.0). The branches, including the tomato sly-miR171 targets, are enlarged. Red arrowheads mark the tomato proteins encoded by the predicted sly-miR171 target mRNAs. (D) Accumulation of predicted sly-miR171 target transcripts in meristematic tissues of tomato cv. M82 based on published RNA-Seq data from (Park *et al.*, 2012). EVM, early vegetative meristem; EVM lp, P1 leaf primordium; FM, floral meristem.

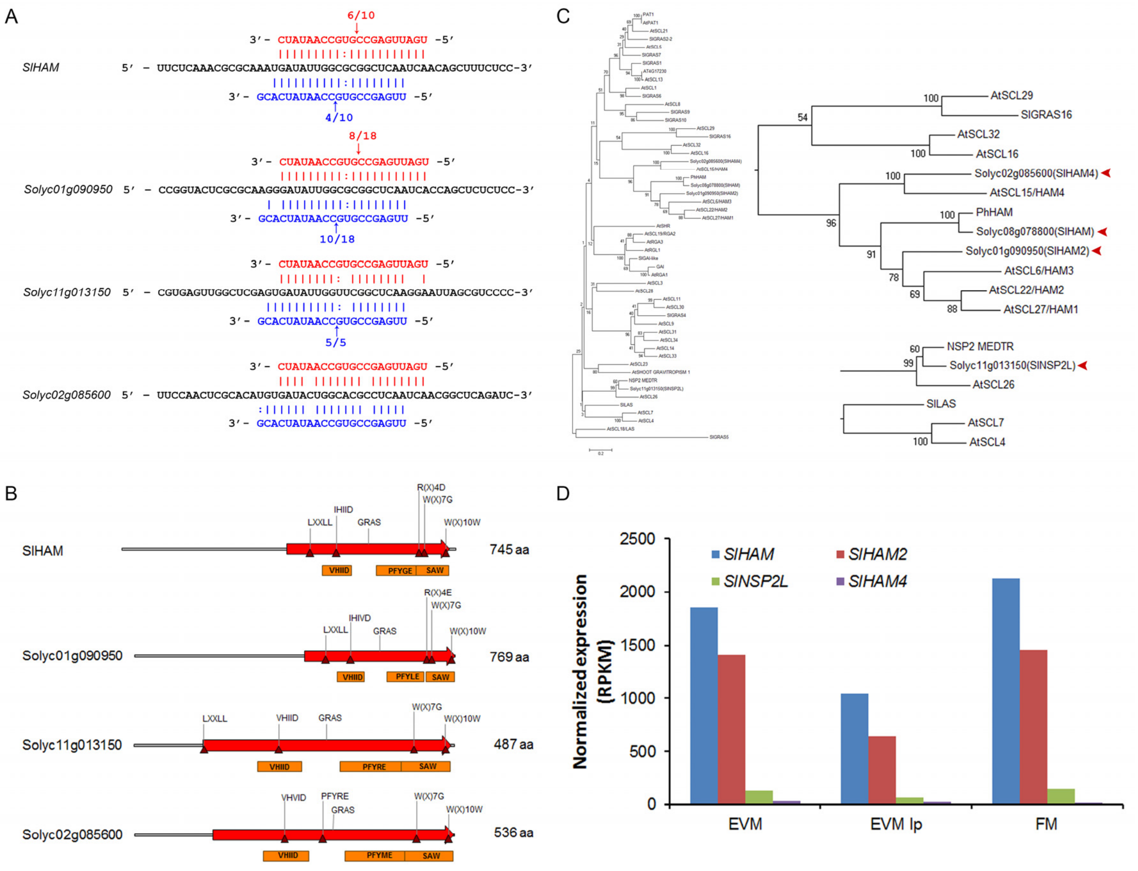

Fig. S2. Generation and screening of transgenic tomato responder lines. (A) Schematic representation of the pART27-OP:SlMIR171a and pART27-OP:SlMIR171b responder binary constructs used for transformation. The respective miR171 sequence is indicated in green. (B) Northern blot analysis of sly-miR171 in 3-weeks- old seedlings. Total RNA (5 µg) was extracted from seedlings of control (*35S:LhG4*) and the indicated transactivated T1 progeny. EtBr staining of tRNA and rRNA served as a loading control. The responder lines, which were used for further analyses are marked by a red circle.

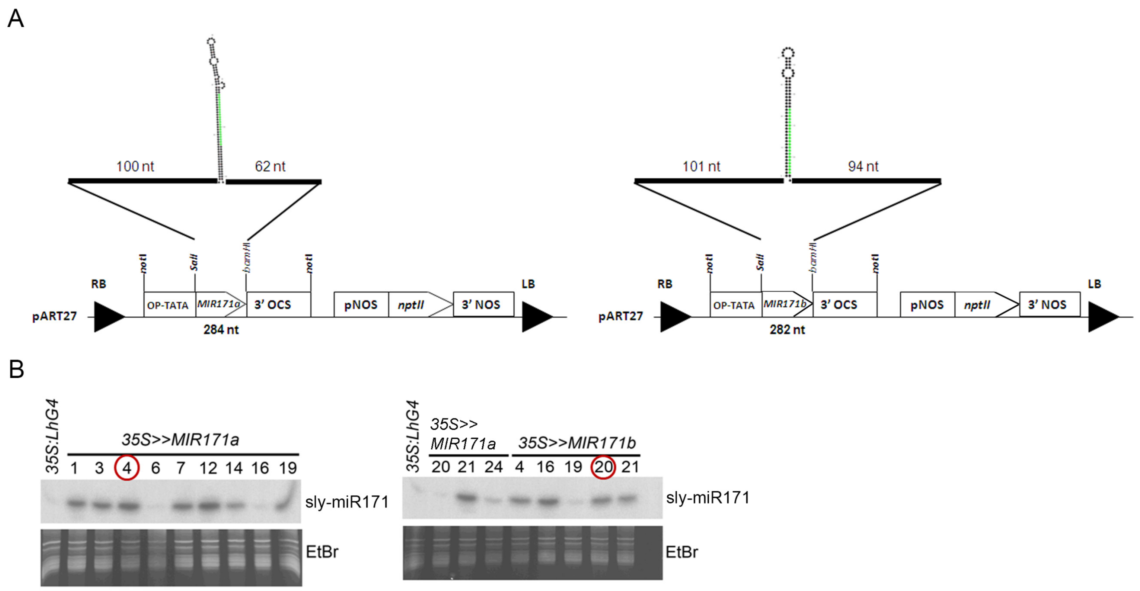

Fig. S3. Phenotype of *35S>>MIR171a* seedlings 25 DAG. The arrow marks the swollen apex. Scale bars = 1 cm.

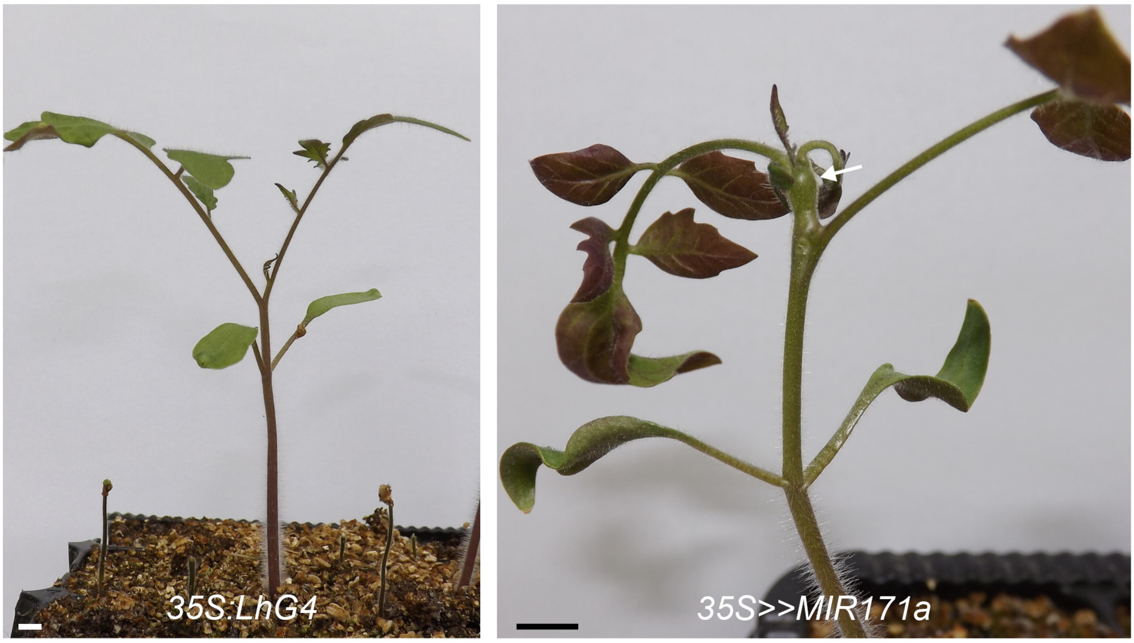

Fig. S4. Characterization of *FIL>>MIR171b* leaves. (A,B) Quantitation of primary and intercalary leaflets of 5th (A) and 10th (B) leaves. Error bars indicate SE (n = 21 from independent plants). Different letters indicate statistically significant differences at P<0.01.

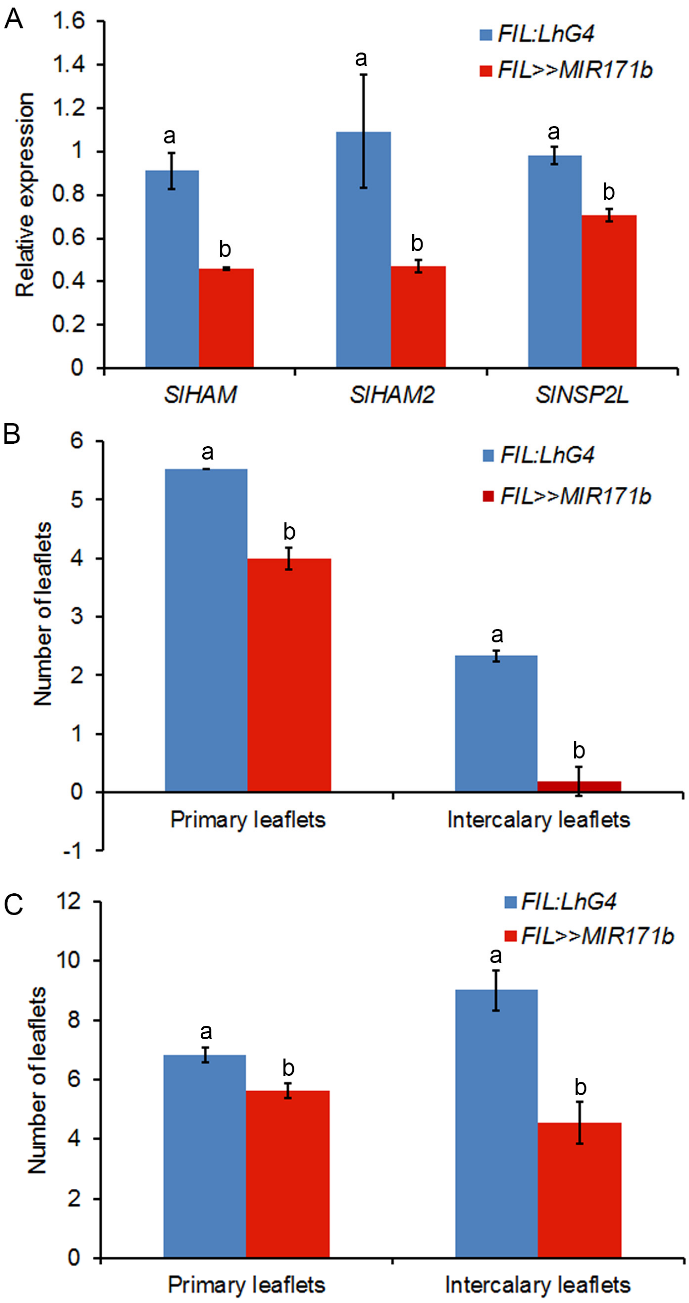

Fig. S5. In-situ hybridization of *SlHAM* and *SlWUS* in *FIL>>MIR171b* floral buds. (A, B) Representative flowers of the indicated genotypes at anthesis. Scale bar = 1

cm. (C-F) expression patterns of *SlHAM* and *SlWUS* in floral bud of the indicated genotypes. s, sepal. Scale bars = 50 µm.

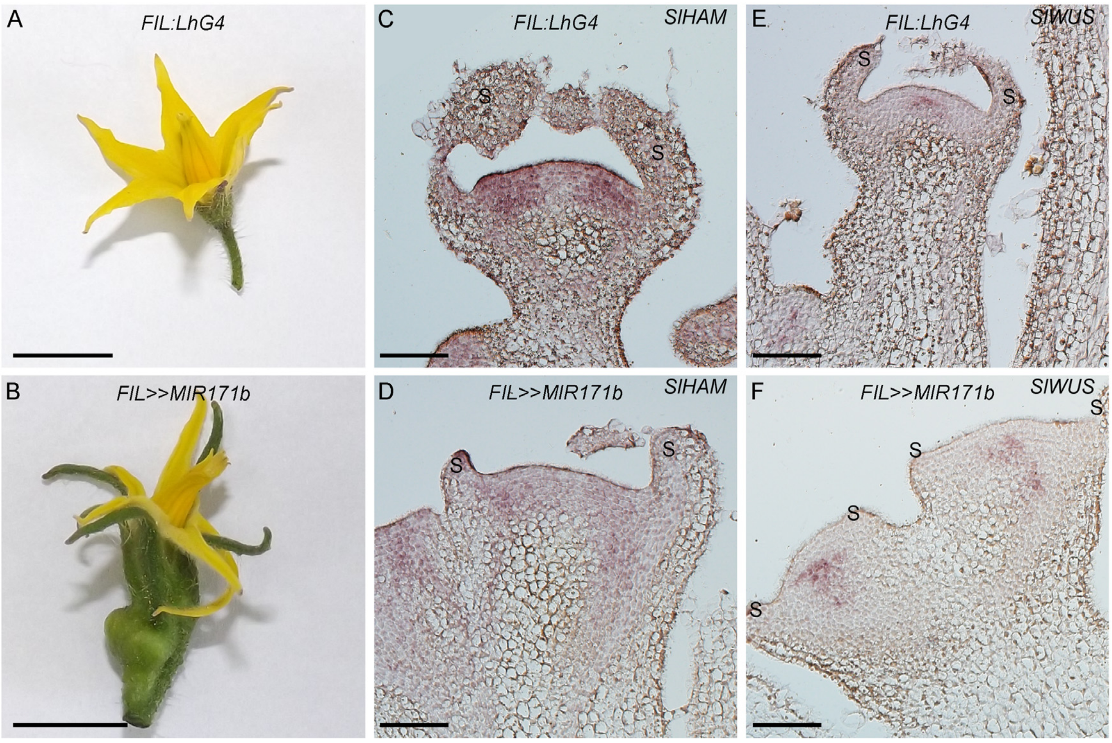

Fig. S6. In-situ hybridization of *SlCLV3* in *SlHAM*s-silenced plants. (A) *SlCLV3* signal was detected in the fully expanded leaf rachis of *FIL>>MIR171b* at the adaxial side region. Ectopic development of leaf primordia (lp) and flower meristem (fm). Scale bar = 100 µm. (B, C) *SlCLV3* expression patterns were detected in the apices of 1.5 DAG seedlings of indicated genotypes. Inset in (C) shows inner section of the same apex. c, cotyledon; lp, leaf primordium; *, SAM. Scale bars = 50 µm. (D, E) *SlCLV3* expression patterns were detected in floral buds of indicated genotypes. s, sepal. Scale bars = 50 µm.

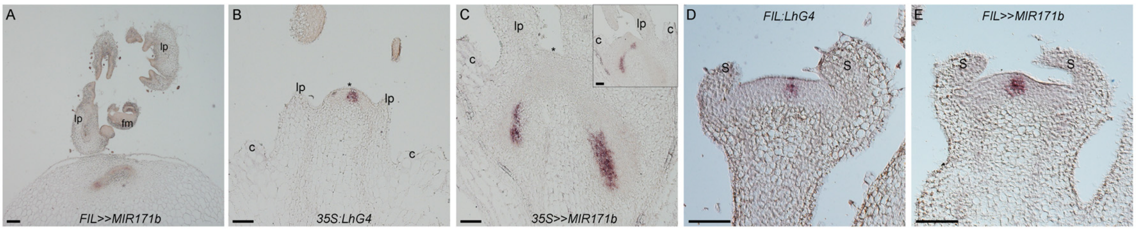

Supplement: Supplementary Data [file supp_erw388_supplementary_table_S1_figures_S6.pdf]
